# Exploiting an Interleukin-15 Heterodimeric Agonist (N803) for Effective Immunotherapy of Solid Malignancies

**DOI:** 10.3390/cells12121611

**Published:** 2023-06-12

**Authors:** Grace Lui, Christine M. Minnar, Patrick Soon-Shiong, Jeffrey Schlom, Sofia R. Gameiro

**Affiliations:** 1 Center for Immuno-Oncology, Center for Cancer Research, National Cancer Institute, National Institutes of Health, Bethesda, MD 20892, USA; grace.lui@nih.gov (G.L.); christine.minnar@nih.gov (C.M.M.); sofia.gameiro@nih.gov (S.R.G.); 2 ImmunityBio, Culver City, CA 90232, USA; pss@nantworks.com

**Keywords:** IL-15, N803, Anktiva^®^, cancer immunotherapy, cytokine

## Abstract

Identifying effective immunotherapies for solid tumors remains challenging despite the significant clinical responses observed in subsets of patients treated with immune checkpoint inhibitors. Interleukin-15 (IL-15) is a promising cytokine for the treatment of cancer as it stimulates NK and CD8^+^ lymphocytes. However, unfavorable pharmacokinetics and safety concerns render recombinant IL-15 (rIL-15) a less attractive modality. These shortcomings were addressed by the clinical development of heterodimeric IL-15 agonists, including N803. In preclinical tumor models, N803 elicited significant Th1 immune activation and tumor suppressive effects, primarily mediated by NK and CD8^+^ T lymphocytes. In addition, multiple clinical studies have demonstrated N803 to be safe for the treatment of cancer patients. The combination of N803 with the immune checkpoint inhibitor nivolumab demonstrated encouraging clinical responses in nivolumab-naïve and nivolumab-refractory patients with non-small cell lung cancer. In a recent Phase II/III clinical study, most Bacillus Calmette–Guerin (BCG)-refractory bladder cancer patients treated with N803 plus BCG experienced durable complete responses. Currently, N803 is being evaluated preclinically and clinically in combination with various agents, including chemotherapeutics, immune checkpoint inhibitors, vaccines, and other immuno-oncology agents. This report will review the mechanism(s) of action of N803 and how it relates to the preclinical and clinical studies of N803.

## 1. Introduction

The success of immune checkpoint inhibition (ICI) in a small subset of cancer patients highlights the promise and challenges of immune therapy for cancer. There is an unmet clinical need for novel therapeutic interventions beyond ICI able to rescue lymphocyte homing and dysfunction in the tumor microenvironment (TME) and to overcome the mechanisms of ICI resistance such as the dysregulation of interferon gamma (IFNγ) signaling, poor major histocompatibility complex I (MHC-I) expression, or defects in antigen processing and presentation (APM). The use of novel immunocytokine delivery strategies and combination therapy approaches may allow for targeting these barriers and increasing the clinical benefit of the treatment of solid malignancies.

Interleukin-15 (IL-15) is a promising immunocytokine for cancer therapy. It is a member of the common receptor gamma chain family together with IL-2, IL-4, IL-7, IL-9, and IL-21. This family of cytokines elicits a broad spectrum of activity in both innate and adaptive immunity, with important clinical implications, which has been reviewed elsewhere [1,2]. Biologically, the IL-15 cytokine is produced as a stable heterodimer encompassing the IL-15 polypeptide single-chain bound to co-expressed IL-15 receptor α (IL-15Rα) which can be cleaved from the cell surface [3,4]. IL-15Rα displays a high affinity for IL-15, functioning not as a receptor but as the specific binding protein to the IL-15 single-chain, allowing the heterodimeric complex to form in the endoplasmic reticulum and enabling its transport to the cell surface as a membrane-bound cytokine [5,6]. IL-15Rα is critical for the bioactivity of this complex as it stabilizes and anchors IL-15, allowing the producing cells to “trans-present” IL-15 to neighboring cells expressing its receptor [4]. A variety of cell types, including blood endothelial cells, lymph node and bone marrow stromal cells, monocytes, macrophages, and dendritic cells, constitutively express IL-15 and IL-15Rα mRNA in a coordinated manner [7,8,9]. In vivo studies using IL-15 reporter mice have demonstrated monocytes, macrophages, and dendritic cells as the main IL-15 producers [10,11].

**Figure 1:**
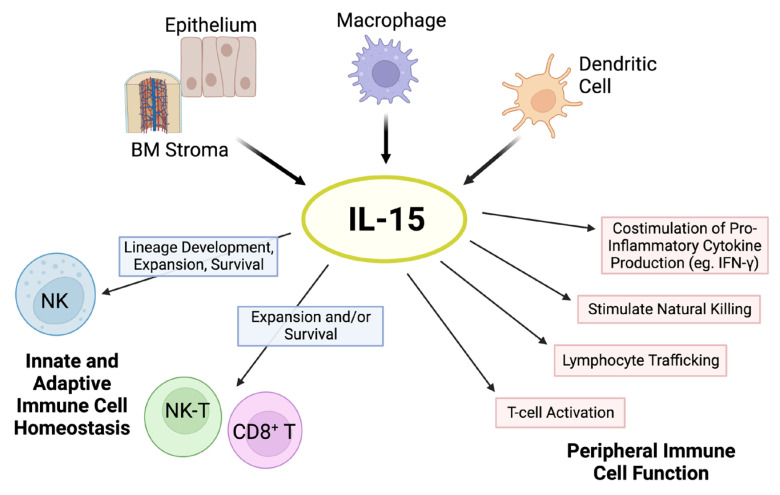
Pleiotropic effects of IL-15. The cytokine IL-15 is produced by multiple cell types, such as activated dendritic cells, monocytes/macrophages, epithelial cells, and bone marrow (BM) stromal cells. IL-15 is critical for natural killer (NK) cell lineage development, survival, and proliferation. This cytokine holds important modulatory effects in the expansion and survival of CD8^+^ T cells, including memory formation, and lymphocyte cytotoxic function. In addition, IL-15 has pleiotropic effects on peripheral innate and adaptive immunity. Schematic adapted from ([23]; Copyright © 2001 American Society of Hematology) and created with BioRender.com.

IL-15 signals through a receptor complex comprising the IL-2/IL-15 receptor β (CD122) and the shared common γ chain subunit (CD132) on immune cells, thereby initiating multiple signaling pathways that support cell expansion and maintenance, with important immune consequences (Figure 1) [12].

The shared IL-2β chain of the IL-15 signaling complex renders some overlapping functions with IL-2, including the induction of T-cell proliferation and cytotoxicity, and natural killer (NK) cell differentiation/activation [13,14]. However, IL-15 is required for NK development, expansion, and functional maturation as shown by the marked reduction in NK cells in IL-15-deficient but not IL-2-deficient mice [15]. While IL-2 can promote T-cell activation-induced cell death (AICD), IL-15 serves as an anti-apoptotic factor for T cells, thereby preventing AICD [16,17,18,19,20]. In addition, IL-15 stimulates the proliferation of memory CD8^+^ CD44^hi^ T cells expressing high levels of the IL-2β chain [13,16,18,20,21,22]. In contrast to IL-2, IL-15 does not support the proliferation, function, and differentiation of regulatory T cells (Tregs) [13,16,18]. An important clinical distinction between these cytokines is the induction of severe capillary leak syndrome by IL-2 therapy, a toxicity not shared by IL-15 [18]. These functional distinctions render IL-15 more favorable than IL-2 as a cancer immunotherapy given its ability to activate immune cells with tumor-suppressive ability and the potential for lower toxicity.

Herein, we review the current knowledge on the use of IL-15 as a cancer therapy, with a particular focus on the mechanism(s) of action and preclinical and clinical development of N803 (Anktiva^®^, formerly known as ALT803), an IL-15 heterodimeric agonist.

## 2. Recombinant IL-15 Studies

Soluble recombinant IL-15 (rIL-15) has been examined in multiple preclinical and clinical studies and has been reviewed in more detail elsewhere [24,25]. Preclinical studies in various murine tumor models demonstrated rIL-15 to elicit significant tumor suppression facilitated by increased NK and CD8^+^ T-cell function. In addition, IL-15 has been reported to protect antigen-activated NKT cells from macrophage-mediated functional suppression, thereby enabling significant tumor control [26]. In combination with monoclonal antibodies targeting CD20 or CD52, rIL-15 further enhanced tumor control, which was associated with increased antibody-dependent cell cytotoxicity (ADCC) mediated by macrophages and/or NK cells [24,27].

In cancer patients, decreased IL-15 expression has been shown to correlate with a lower proliferation of B and T cells, a higher risk of tumor recurrence, and decreased patient survival [28]. In the first in-human study, metastatic patients with melanoma and renal cell carcinoma (RCC) treated with intravenous (i.v.) recombinant human IL-15 (rh-IL15) displayed an expansion of NK cells, plus memory and γδ T cells [29]. Whereas IL-15 induced NK cell proliferation, the expansion rate of CD56^bright^ NK cell populations exceeded that of CD56^dim^ NK cells. Notably, CD56^bright^ NK cells gained cytotoxic ability against tumor cells to levels comparable to those of CD56^dim^ populations [30]. Whereas no objective responses were observed, the elimination of lung lesions was observed in two melanoma patients [29]. However, rhIL-15 demonstrated a short half-life (~2.5 h) and dose-limiting toxicities at the doses required for clinical benefit. The maximum-tolerated dose (MTD) was 0.3 μg/kg [29]. Subsequent clinical studies in which rIL-15 was administered subcutaneously (s.c.) allowed for a significant increase in dose and MTD, with NK cell expansion observed at all dose levels [31]. While disease stabilization was observed in renal cell carcinoma and non-small cell lung cancer (NSCLC) patients, objective clinical responses were not attained. However, patients treated at the higher dose levels experienced dose-limiting toxicities including grade 2–3 fevers and grade 3 chest pain in one patient [8]. IL-15 is currently being evaluated in multiple clinical studies in combination with other immuno-oncology agents, including immune checkpoint inhibitors and monoclonal antibodies [32].

## 3. IL-15 Heterodimeric Agonists

Due to its instability, the clinical application of soluble rIL-15 is hindered by its short half-life, spanning from ~2.5 h when administered i.v. to ~12 h upon s.c. administration [29,31]. To overcome these limitations, several IL-15/IL-15Rα heterodimeric agonists have been developed. These agents display a higher stability and potency relative to soluble rIL-15 and have been demonstrated to not require the IL-15 presentation in *trans* to induce immune activation. Some of these novel IL-15 agonists are in clinical development and are reviewed elsewhere [33,34]. Here, we describe the mechanism(s) of action and preclinical and clinical status of N803, the soluble IL-15/IL-15Rα heterodimeric agonist most advanced in clinical development.

## 4. N803

**Figure 2:**
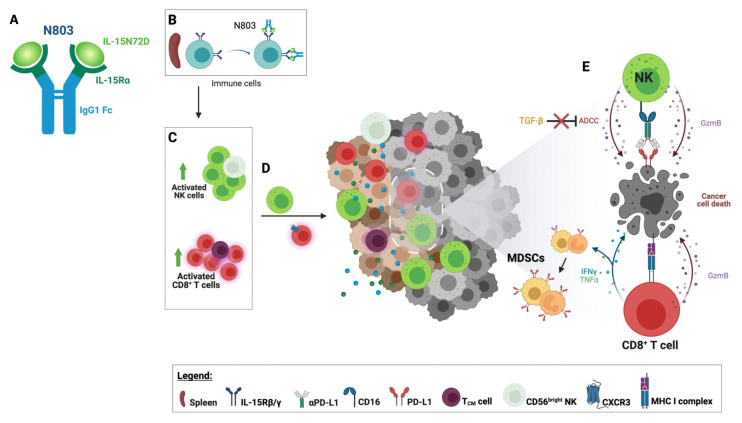
Immune effects of N803. (**A**) N803 comprises mutated (N72D) human IL-15 bound to the IL15Rα fused with a human IgG1 Fc. (**B**) N803 binds to circulating immune cells through the IL-15 receptor. (**C**) The binding of N803 to lymphocytes leads to the activation and expansion of natural killer (NK) cells and CD8**^+^** T-cell populations, resulting in the expansion of high effector CD56^dim^ and CD56^bright^ NK cells and central memory T cells (T_CM_). (**D**) The upregulation of CXCR3 on the surface of splenic CD8^+^ T cells increases the potential for homing into the tumor microenvironment (TME). Activated NK cells also migrate to the tumor. (**E**) Activated tumor-infiltrating CD8**^+^** T lymphocytes (TILs) recognize cancer cells through recognition of the tumor-associated antigen epitopes presented by the MHC class I complex. Activated CD8**^+^** T cells and NK cells display enhanced cytotoxicity leading to cancer cell death. CD8^+^ T cells release Th1 cytokines such as IFNγ and TNF⍺, promoting an inflamed TME and upregulating PD-L1 on granulocytic and monocytic myeloid-derived suppressor cells (MDSCs). N803 renders NK TILs resistant to the effects of TGF-β and capable of enhanced antibody-dependent cellular cytotoxicity (ADCC). Figure created with BioRender.com.

N803 is a novel mutated (N72D) human IL-15 (IL-15N72D) bound to a dimeric IL-15Rα Sushi domain and an IgG1 Fc fusion protein forming a stable heterodimeric complex (Figure 2A). This complex was designed to increase IL-15′s stability and half-life while mimicking its endogenous biology. Heterodimerization of the IL-15 chain with IL-15Rα has been shown to preserve IL-15 chain stability [35]. Moreover, the IL-15 asparagine to aspartic acid substitution lends N803 a 4–5-fold increase in biological activity compared to single-chain wild-type IL-15 via increased binding to IL2Rβ and agonism of the IL2Rβ/IL2Rγ receptor [36,37]. In vitro studies in the presence of soluble human IL-15Rα-Fc reported the N72D IL-15 mutation to confer ~3.4-fold increase in biological activity relative to rhIL-15, suggesting increased biological activity of the hIL-15N72D heterodimer versus native heterodimeric IL-15 [37]. It is possible that heterodimerization and the N72D mutation may add to N803 stability through decreased IL-15 deamidation [38]. N803 is a potent inducer of the activation, proliferation, and cytolytic function of NK cells and CD8^+^ T cells, eliciting pleiotropic immune effects supportive of tumor suppression as a monotherapy and in combination with other agents (Figure 2).

### 4.1. N803 as Single Agent

The safety, pharmacokinetics, and immune modulatory effects of N803 were first studied in murine hosts and cynomolgus monkeys [36,39,40]. In mice, N803 exhibited a 35-fold higher half-life relative to rhIL-15, allowing for a longer stimulation of NK and CD8^+^ T cells [37,41]. In cynomolgus monkeys, N803 displayed a half-life of ~8 h, inducing a wide dose-dependent expansion of peripheral blood lymphocytes after intravenous administration without increased IL-2, IFNγ, TNFα, IL-4, IL-5, or IL-6 plasma levels. No toxicity was observed [41].

In murine tumor models, N803 has demonstrated significant tumor suppression and/or anti-metastatic effects against multiple transplanted murine solid tumors such as the 4T1 breast, B16F10 melanoma, CT26 colon carcinoma, and GL261-luc glioblastoma models [39,41,42]. Antitumor effects have also been reported in carcinogen-induced orthotopic non-muscle invasive bladder tumors [43]. The ability of N803 to induce antitumor effects stems from its ability to induce significant expansion and activation of NK cells and CD8^+^ central memory T cells in the periphery, as well as IFNγ production and infiltration of CD8 T cells into the tumor microenvironment [41,42].

The regulation of NK and T-cell populations by N803 has been associated with the preferential stimulation of Th1 versus Th2 cytokines. In vitro, N803 has been shown to induce IFN-γ production by human peripheral blood mononuclear cells (PBMCs) without modulating anti-inflammatory and immune suppressive cytokines, such as IL-4, IL-10, or IL-17A [41]. In mice, N803 induced an 11-fold increase in IFN-γ 1 day post-dosing, followed by ≤5-fold increases in TNF-α, IL-5, IL-6, and IL-10 [39]. The proliferative effects of N803 appear more pronounced in NK cell populations and innate memory CD8^+^ T cells expressing NKG2D [39]. N803 has been shown to increase the lytic ability of the human herpes virus 16 (HPV16) E7-specific cytotoxic CD8^+^ T cells in vitro against HPV16^+^ carcinoma targets, including after target exposure to sublethal radiation [44].

N803 exposure modulates murine and human NK cells in multiple and comparable ways. N803 promotes the development of highly cytolytic murine CD11b^+^ CD27^high^ NK cells, with increased cytokine production and migratory capacity [39]. The induction of this high-effector NK cell population was associated with anti-metastatic activity in the lung elicited by N803 in a 4T1 orthotopic breast cancer model [39]. Similarly, N803 has been demonstrated to increase the lytic ability of human NK cells to lyse a spectrum of triple-negative breast (TNB) cancer cell lines in vitro, further amplified upon tumor target exposure to the estrogen receptor antagonist fulvestrant. These effects translated to a significant suppression of 4T1 tumors in immune-competent mice [45]. In addition, N803 has been shown to protect human NK cells from the immunosuppressive effects of transforming growth factor beta (TGFβ), rescuing their cytolytic function through the inhibition of TGFβ1 signaling and Smad2/3-induced gene transcription [46]. Additional evidence of N803 affecting the development of NK cells can be seen from the priming of human CD56^bright^ NK cells, conventionally considered immature and associated with immunomodulatory effects. However, upon in vitro exposure to N803, CD56^bright^ NK cells displayed increased cytokine production, cytotoxicity, and degranulation, indicating an enhancement of tumor-suppressing ability [47]. These findings were demonstrated in vivo where NSG mice inoculated with N803-primed human CD56^bright^ NK cells experienced better control of the K562 leukemia tumor burden [47]. N803 also demonstrated similar effects on the expansion and lytic ability of human NK cells against leukemia and GD2^+^ pediatric solid tumor xenografts [48,49]. Recent studies have demonstrated the potential of N803 as a therapeutic for the treatment of small cell lung cancer (SCLC). In vitro studies demonstrated that exposure to N803 enabled NK cells to efficiently lyse a spectrum of human SCLC cell lines irrespective of their molecular subtype and MHC I expression. These effects translated into effective tumor control in a human SCLC xenograft model [50].

### 4.2. N803 in Combination Therapy

N803 has been shown to increase the cell-surface expression of programmed cell death ligand 1 (PD-L1) in immune cells infiltrating murine TNB 4T1 tumors, particularly granulocytic and monocytic myeloid-derived suppressor cells (MDSCs) via IFNγ induction [41,51]. Combination therapy with N803 and αPD-L1 monoclonal antibody (mAb) has been evaluated in both “warm” (MC38-CEA colon) and “cold” (4T1 breast) murine tumor models [51,52]. In contrast to either monotherapy, N803 plus αPD-L1 reduced 4T1 lung metastasis and MC38-CEA tumor burden, resulting in survival benefit [51]. These effects were associated with increased activation, proliferation, and cytotoxicity of CD8^+^ T cells and NK cells, both determinants of tumor suppression [51]. Consistent with these findings, N803 has been shown to augment NK cell-mediated ADCC of human carcinoma in the presence of mAbs targeting PD-L1 [48,53]. Notably, these cytotoxic effects elicited by N803 resulted in efficient lysis of cancer-stem cell-rich chordoma cell lines in vitro via mAbs targeting PD-L1 and the epidermal growth factor receptor (EGFR) [54]. The stimulatory effects mediated by N803 have also been observed in human xenograft models of oral cavity squamous carcinomas, where the addition of N803 to the combination of a PD-1-targeting mAb plus adoptive transfer of an irradiated human NK cell line harboring a PD-L1-specific chimeric antigen receptor (CAR) elicited synergistic tumor control [55].

Preclinical studies in MC38 expressing CEA colon and 4T1 TNB cancer models examined the effects of N803 in combination with the class I histone deacetylase (HDAC) inhibitor entinostat plus adenovirus-based vaccines targeting the pan-cancer carcinoembryonic antigen (Ad-CEA) or Twist1 (Ad-Twist1), a metastasis-associated transcription factor [56]. Whereas N803 in combination with vaccine promoted circulating activated CD8^+^ T cells with augmented expression of the trafficking chemokine receptor CXCR3, CD8^+^ T cells showed poor tumor infiltration and granzyme B expression in the TME, resulting in modest antitumor efficacy. In contrast, triple combination therapy promoted significant tumor control versus monotherapies or doublet combinations in both tumor models, and a significant decrease in the number of 4T1 lung metastases. These effects were primarily dependent on CD8^+^ T cells, whose activity was significantly enhanced by N803 and the vaccine. In the periphery, the N803 component drove CD8^+^ T cells’ expansion, activation, and memory formation. Further, N803 plus vaccine expanded CD8^+^ T cells, producing IFNγ and/or TNFα in the tumor microenvironment and the periphery. At the tumor site, N803 in combination with the vaccine alone or plus entinostat induced a significant Treg reduction and increased the activation of CD8^+^ T lymphocytes (TILs), including those expressing granzyme B. The synergistic effects of triple combination resulted in significant T-cell responses to vaccine and cascade antigens, including neoepitopes, the maximal infiltration of CD8^+^ T cells, as well as the enhanced transcription of genes associated with tumor inflammation and T-cell chemotaxis, including the CXCR3 ligands CXCL9, CXCL10, and CXCL11.

N803 has been evaluated preclinically in multiple-agent combinations [52,57]. N803 in combination with the Ad-(CEA or Twist1) vaccine was included in a hexatherapy regimen comprising docetaxel and mAbs targeting OX40, 4-1BB, and PD-L1 [52]. Hexatherapy synergized to provide superior CD8^+^ T-cell-dependent suppression of 4T1 and MC38-CEA tumors and 4T1 lung metastasis, compared to monotherapies or other agent combinations. These effects were associated with the increased activation and tumor infiltration of CD8^+^ T cells, decreased MDSC populations, and induction of antigen-specific T cells and antigen cascade responses. Within these dynamic effects of hexatherapy, N803 contributed to the increased proliferation and decreased exhaustion of CD8^+^ T cells in the TME, as well as the enhanced transcription of tumor IFNγ and T-cell-attracting chemokines.

Preclinical studies targeting MC38-CEA, 4T1, and LLC (lung) tumors explored the ability of N803 to activate tumor-specific T cells in combination with Ad-(CEA or Twist1), OX40, and GITR agonists, and the indoleamine 2,3-dioxygenase (IDO) inhibitor epacadostat [57]. Tumor therapy with N803 and the vaccine elicited immune-mediated tumor control associated with the enhanced expression of co-stimulatory molecules on immune populations. However, pentatherapy induced the highest tumor suppression via effector T-cell activation and decreased tumor immunosuppression, consistent with the observed protective immunological memory. In a murine model of bladder cancer, the intravesical administration of N803 in combination with BCG treatment induced tumor suppression associated with augmented tumor infiltration of activated NK cells and CD8^+^ tumor-infiltrating lymphocytes [58].

## 5. Clinical Studies Involving N803

### 5.1. Phase I Clinical Studies

**Figure 3:**
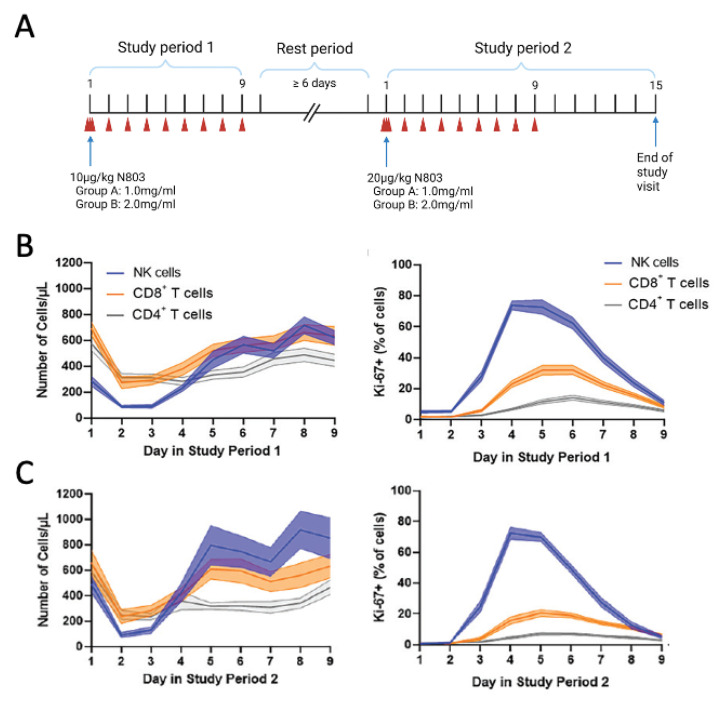
N803 favors the expansion of natural killer (NK) cells and CD8^+^ T cells in healthy volunteers. (**A**) Study design [59]. N803 was administered subcutaneously at 10 μg/kg on day 1 of study period 1 and 20 μg/kg on day 1 of study period 2. Subjects in group A and group B were administered 1.0 and 2.0 mg/mL N803, respectively. Blood samples for pharmacokinetic analysis were collected (red arrowheads) over nine consecutive days after each N803 administration. On day 1 in both study periods, blood samples were collected before N803 administration, and at 1 and 4 h after administration. (**B**,**C**) The number of CD8^+^ T cells, CD4^+^ T cells, and NK cells per μL of blood and respective Ki67 expression in (**B**) study period 1 and (**C**) study period 2. Graphs show the mean values (lines) and S.E.M. (shading). All panels were adapted from [59]; Copyright 2022. The American Association of Immunologists, Inc.

In the first in-human study, 20 healthy volunteers were dosed with N803 subcutaneously at 10 µg/kg on the first day of study period 1 and were followed for 8 additional days; after a rest period of at least 6 days, study period 2 was initiated, where 14 of these subjects received an additional dose of N803 (s.c.) at 20 µg/kg on the first day (Figure 3A). N803 demonstrated a half-life of ~20 h, over 20-fold longer than rhIL-15. N-803 was well tolerated and no serious adverse events (AEs) or grade ≥ 3 AEs were observed [59]. The most common AEs included injection site reactions, chills, and pyrexia. N803 induced sustained increases in NK cell numbers and Ki67 expression in both NK cells and CD8^+^ T cells at both dose levels (Figure 3B,C). This and subsequent Phase I studies in cancer patients demonstrated the subcutaneous route to be clinically favorable to N803 relative to intravenous delivery as it enabled the reduced severity and number of adverse events. Moreover, s.c. administration enabled sustained N803 serum concentrations, eliciting NK cell expansion correlating with significant increases in serum IFNγ and TNFα [60,61,62].

In the initial Phase I study in patients with advanced solid tumors, where total lymphocyte and CD8^+^ T-cell expansion were mild, significant NK cell expansion was observed. N803 was well tolerated, with low-grade AEs such as nausea and fatigue most observed in patients dosed intravenously. The most common AE reported upon N803 subcutaneous delivery was pain at the injection site. No clinical activity was noted [60]. To date, clinical studies indicate N803 to be well-tolerated at doses up to 20 μg/kg (MTD not reached), with mild and non-dose limiting toxicities [60,61,62]. N803 is currently being evaluated in multiple clinical studies in combination with standard-of-care (SOC) therapies and additional immuno-oncology agents for the treatment of solid malignancies (Table 1).

### 5.2. N803 in Combination with Immune Checkpoint Inhibitors and Other Immuno-Oncology Agents

**Figure 4:**
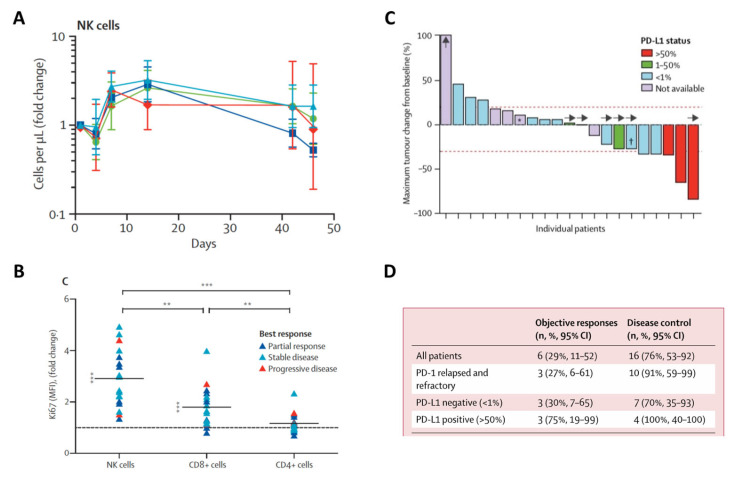
Immune correlates (**A**,**B**) and clinical effects (**C**,**D**) in cancer patients treated with N803 plus nivolumab**.** Fold change versus baseline in (**A**) peripheral natural killer (NK) cell numbers and (**B**) Ki67 expression (MFI) in NK and T–cell subsets on day 4. Each symbol represents one patient, with colors denoting the best response as depicted in the graph inset. (**C**) The best clinical response attained in patients treated with combination therapy using nivolumab at 3 mg/kg followed by a 240 mg flat dose. Upward arrow denotes one patient with 180% increase in tumor lesion size per RECIST criteria. Right arrows denote patients with ongoing study treatment. (**D**) Objective responses and disease control rates. Figure adapted from ([62]; Copyright 2018, with permission from Elsevier). * Progression due to a new lesion. † Target lesion decreased 27% but met RECIST 1.1 criteria for partial response. ** *p* < 0.001. *** *p* < 0.0001.

A Phase 1b clinical study (NCT02523469) examined combination therapy with N803 and the αPD-1 mAb nivolumab in patients with advanced NSCLC. The analysis of peripheral blood demonstrated that the combination therapy induced significant NK cell expansion and Ki67 expression in NK cells and CD8^+^ T cells. In contrast, no significant effects were observed in peripheral CD4^+^ T cells (Figure 4A,B). Immune activation translated to variable IFNγ responses, associated with the development of transient fever and flu-like symptoms. Combination therapy was well-tolerated, in the absence of dose-limiting toxicities. Injection-site reactions and flu-like symptoms were the most common AEs. Two patients experienced grade 3 lymphocytopenia and two others grade 3 fatigue. One patient experienced a grade 3 myocardial infarction. No grade 4 or 5 toxicity was observed. Clinical responses were observed in 17 of the 21 evaluable patients, irrespective of tumor PD-L1 expression (Figure 4C). A total of 9 patients (43%) experienced a decrease in the target lesion size and 16 (76%) achieved disease control, for an objective response rate of 29% [62]. In this study, 11 patients had previously progressed on αPD-1 therapy. Of these, 10 (91%) experienced disease control, with 3 (27%) achieving a partial response and 7 (64%) experiencing a stable disease (Figure 4D) [62]. In one report, a patient with Merkel cell carcinoma who progressed on SOC achieved a complete response (CR) upon treatment with the αPD-L1 avelumab and nab-paclitaxel; the CR was maintained with continued N803 plus avelumab therapy [63]. Additional clinical studies are currently evaluating N803 in combination with nivolumab and other immune checkpoint inhibitors across multiple solid tumor types.

### 5.3. Clinical Application of N803 in Bladder Cancer

Non-muscle-invasive bladder cancer (NMIBC) comprises over 75% of bladder cancers. Up to 30% of these patients are unresponsive to SOC with intravesical BCG. Of those who initially respond, up to 70% have disease recurrence or become refractory to BCG, after which there are limited treatment options [43,64]. Various therapies are being explored for the treatment of NMIBC [64,65]. 

**Figure 5:**
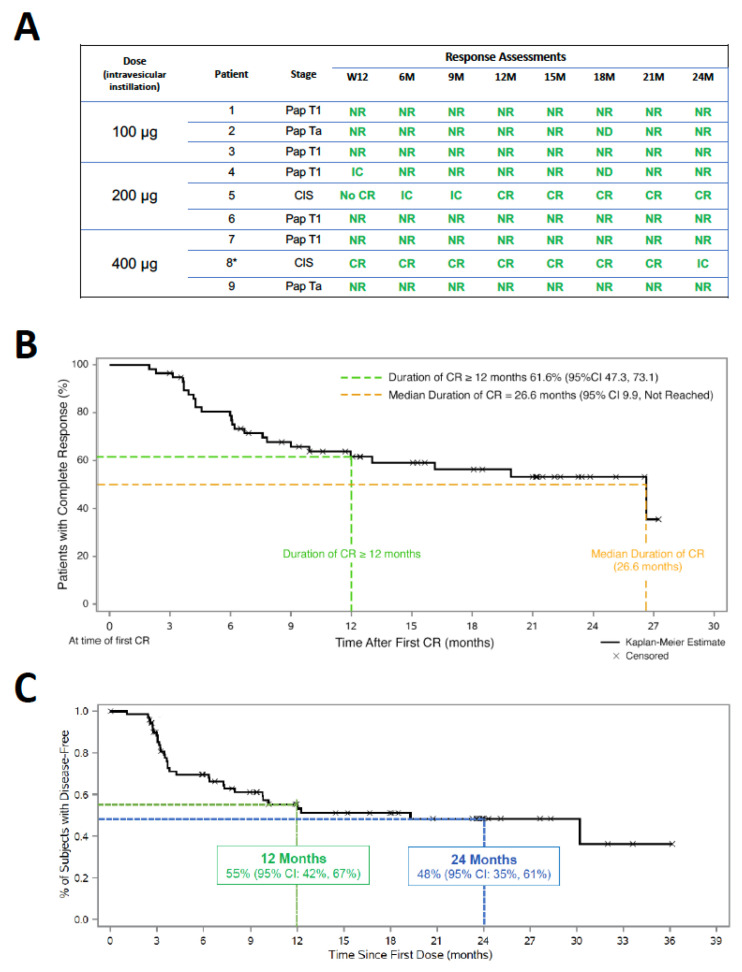
Clinical responses to intravesical N803 plus Bacillus Calmette–Guerin (BCG) therapy in non-muscle-invasive bladder cancer (NMIBC) patients treated in Phase Ib (**A**) and Phase II/III (**B**,**C**) clinical studies. (**A**) N803 dose, patient characteristics, and therapy response in nine patients treated in the Phase Ib study [66,67]. CR: complete response; NR: no recurrence; No CR: no complete response; IC: inconclusive; * only patient to experience disease recurrence. Noted at 38 months (carcinoma in situ (CIS)) and treated off study with N803 plus BCG, followed by maintenance BCG. After additional 28 months of follow-up, the patient was disease-free with intact bladder. (**B**,**C**) Duration of complete response in CIS (**B**) and papillary (pap) (**C**) disease patients treated with N803 and BCG in the Phase II/III clinical study [68]. Panel **A** adapted from ([67]; © 2021 Rosser et al. Published with license by Taylor & Francis Group, LLC). Panels (**B**,**C**) adapted from [68]; see also Chamie et. al., NEJM Evid 2023, DOI: 10.1056/EVIDoa2200167.

A Phase 1b clinical study examined intravesical N803 administration in association with BCG for the treatment of BCG-naive patients (NCT02138734). No dose-limiting toxicities were noted, with all adverse events below grade 3. BCG alone resulted in a response rate of 50%. In contrast, all patients treated with N803 combination therapy were disease-free at 2 years of follow-up (Figure 5A). Six years after this combination therapy, all patients had intact bladders and no disease recurrence [66,67]. A phase II/III clinical study (NCT03022825) examined this combination therapy in 160 patients with BCG-unresponsive carcinoma in situ (CIS, n = 83) and papillary (n = 77) NMIBC. Approximately 71% (59/83) of the CIS patients treated with combination therapy had a complete response, with a median duration of 26.6 months (Figure 5B). Patients with papillary disease achieved a disease-free survival rate of 55% at 12 months and 48% at 24 months (Figure 5C). Cystectomy was avoided in 96% of CIS and 95% of papillary NMIBC-responsive patients at 24 months of follow-up. Bladder cancer-specific overall survival was 99% at 24 months of follow-up. This clinical study demonstrated the efficacy and safety profile of intravesical N803 plus BCG to exceed that of other treatment options for BCG-unresponsive non-muscle invasive bladder cancer [68]. Based on these clinical responses, a biologics license application (BLA) for the use of intravesical N803 in combination with BCG in NMIBC was recently submitted to the Food and Drug Administration (FDA).

## 6. Discussion

The pleiotropic and unique biology of IL-15 render this cytokine very attractive for the agnostic treatment of cancer. Given the safety challenges of recombinant IL-15, the pioneering development of N803 has enabled safe administration to patients and the clinical evaluation of the unrealized potential of IL-15 as a cancer therapeutic [24,59,60]. Furthermore, preclinical and clinical studies have demonstrated that N803 induces tumor control while overcoming the short half-life of recombinant IL-15 [24,61,69]. Importantly, both intravenous and subcutaneous N803 administration have demonstrated an improved safety profile relative to rIL-15, including no reported dose-limiting toxicities [29,31]. Intravenous delivery allows for systemic and broad cytokine distribution to off-target sites and can result in high cytokine levels, collectively amenable to the development of immune-related AEs. In contrast, subcutaneous delivery may enable lymphatic distribution conducive to the targeted delivery to lymph nodes upstream of tumor lesions, where the most effective dendritic cell priming of T cells may occur. While N803 was shown to be safely administered to cancer patients by either route, subcutaneous dosing demonstrated a more favorable pharmacokinetic profile, with lower and more sustained N803 blood levels, resulting in a safety profile with minimal immune-related systemic toxicities at clinically-active doses [60].

N803 has also demonstrated the ability to mediate hallmark IL-15 immune effects in preclinical and clinical studies. Its increased agonism of the IL-15 receptor and the prolonged stimulation of both NK and CD8^+^ T cells enables the durable proliferation, activation, memory generation, and increased lytic ability of these immune effectors. In addition, the N803 induction of Th1 responses and IFNγ production in the absence of Treg expansion attribute this first-in-class heterodimeric IL-15 agonist with unique promise as a cancer therapeutic. In both mice and cancer patients, the immune-enhancing effects of N803 have translated into significant antitumor effects, particularly in combination with other immuno-oncology agents [34,39,62,63]. In a Phase I clinical study (NCT02523469) in which 21 NSCLC patients received N803 in combination with nivolumab, 76% experienced disease control [62]. The clinical activity observed with this combination was most striking in patients who had previously progressed on αPD-1 therapy, as 91% experienced disease control, 27% had a partial response, and 64% experienced disease stabilization. Despite the limited number of patients in this trial, these findings suggest N803 can re-sensitize refractory patients to αPD-1 therapy. These findings are consistent with the observed expansion and activation of peripheral NK and CD8^+^ T cells, associated with the increased expression of the proliferation marker Ki67 and elevated serum IFNγ [62]. However, whereas Ki67 elevation was observed in most patients attaining an objective response, it was also present in some patients with progressive disease, suggesting that other factors may be at play. T-cell exhaustion, poor NK and/or CD8^+^ T-cell infiltration into the tumor and/or the presence of immunosuppressive entities in the TME hampering lymphocyte function could be associated with disease progression despite strong elevations in Ki67 and the peripheral expansion of immune effectors. Notably, the lower Ki67 expression and dampened IFNγ levels correlated with milder inflammatory AEs, such as fever and flu-like symptoms [62].

Following the positive antitumor effects in preclinical models of bladder cancer [43,58], a Phase Ib clinical (NCT02138734) study in which nine BCG-naïve NMIBC patients received intravesical N803 plus BCG demonstrated safety as well as durable complete responses in all patients treated with the combination therapy [66]. A recent Phase II/III trial (NCT03022825) extended this therapy to NMIBC BCG-refractory patients, demonstrating 71% of CIS and 48% of papillary disease patients experiencing durable complete responses and ≥95% of all patients with cystectomy avoidance at 24 months of follow-up [68].

In future studies, it will be important to investigate the optimal N803 dosing and schedule to minimize toxicity and balance its tumor-suppressive effects with the potential induction of T-cell exhaustion. In addition, studies incorporating agents promoting tumor targeting and antigen specificity may contribute to minimizing off-target immune cell activation and adverse events. Future clinical investigations exploring the associations between the HLA restrictions and gut/skin microbiome with the most effective N803 outcomes may inform the patient selection and discovery of novel combination therapies.

## 7. Conclusions

Mounting preclinical and clinical evidence strongly support the continued clinical development of N803. N803 is a novel and potent Th1-inducing immunocytokine eliciting significant immune effects. Both preclinical and clinical studies have demonstrated that N803 promotes significant immune activation, proliferation, and cytolytic capacity of NK cells and CD8^+^ T cells. Mounting evidence supports the use of N803 in combinatorial approaches, including those involving the inhibition of immune suppressive entities in the tumor microenvironment as promising strategies with potential for clinical benefit. The lack of response of many cancer patients to immune checkpoint inhibitors underscores the unmet clinical need to develop novel therapies applicable to both immune checkpoint-naïve and -refractory patients. To date, results from multiple preclinical studies and a limited number of patients indicate that N803 elicits tumor-suppressive effects that can be further augmented in a combinatorial regimen.

## Figures and Tables

**Table 1:** Selected ongoing clinical studies utilizing N803 for the treatment of solid malignancies.

Indication	Combination Therapy	Stage	Phase	Trial Identifier
Bladder cancer	BCG	BCG-unresponsive non-muscle invasive	Phase II/III	NCT03022825
	BCG	Non-muscle invasive	Phase I/II	NCT02138734
Breast cancer	Multiple SOC and immunotherapies	TNBC after SOC	Phase I/II	NCT03387085
	Sacituzumab, PD-L1 t-haNK, and SOC	TNBC after prior therapies	Phase I/II	NCT04927884
Colorectal cancer	Multiple SOC and immunotherapies	Metastatic	Phase I/II	NCT03563157
Colorectal and small bowel cancers	CV301, M7824, and NHS-IL12		Phase II	NCT04491955
Gastroesophageal junction cancers and advanced HNSCC	Pembrolizumab and PD-L1 t-haNK	Recurrent or metastatic	Phase II	NCT04847466
Head and neck cancer	M7824 and TriAd vaccine (CEA/MUC1/Brachyury)	Resectable and not HPV associated	Phase I/II	NCT04247282
HNSCC	CIML NK cell infusion, and Ipilimumab	Recurrent	Phase I	NCT04290546
Non-small cell lung cancer	Nivolumab	Advanced or metastatic	Phase I/II	NCT02523469
	Pembrolizumab and multiple SOC	Stage 3 or 4	Phase III	NCT03520686
	Pembrolizumab and multiple SOC	Advanced, recurrent, or Stages 3–4B	Phase II/III	NCT05096663
Lung (NSCLC/SCLC), urothelial, HNSCC, MCC, melanoma, RCC, gastric, cervical, hepatocellular, MSI, dMMR, and colorectal	Pembrolizumab, Nivolumab, Atezolizumab, Avelumab, Durvalumab, and PD-L1 t-haNK	Prior immune checkpoint inhibitors	Phase II	NCT03228667
Solid tumors	M-CENK	Advanced or metastatic	Phase I	NCT04898543
Pancreatic cancer	PD-L1 t-haNK, SBRT, and additional SOC	Locally advanced or metastatic	Phase II	NCT04390399
Prostate cancer and solid tumors	M7824, MVA/FP-Brachyury, and Epacadostat	Advanced or metastatic	Phase I/II	NCT03493945

Trials registered at www.clinicaltrials.gov listed with NCT identifiers; access date: 23 February 2023. BCG: Bacillus Calmette–Guerin; CV301: MVA/FP-CEA/MUC1/TRICOM modified Vaccinia Ankara (MVA), triad of co-stimulation molecules ICAM-1, LFA-3, B7-1 (TRICOM); FP, fowlpox; HNSCC: Head and neck squamous cell carcinoma; M7824: bintrafusp alfa, a bifunctional αPD-L1/TGFβRII fusion protein; MCC: Merkel cell carcinoma; M-CENK: memory cytokine enriched NK cells; MSI: microsatellite instable; dMMR: Mismatched repair deficient; NSCLC: non-small cell lung carcinoma; PD-L1-t-haNK: PD-L1 targeting high-affinity NK cells; RCC: Renal cell carcinoma; SBRT: stereotactic body radiation therapy; SCLC: small cell lung carcinoma; SOC: standard-of-care; and TNBC: triple-negative breast cancer.

## Data Availability

Not applicable.
